# The effect of remimazolam on postoperative memory retention and delayed regeneration in breast surgery patients: Rationale and design of an exploratory, randomized, open, propofol-controlled, single-center clinical trial: A study protocol

**DOI:** 10.1097/MD.0000000000027808

**Published:** 2021-12-03

**Authors:** Kazuhiro Shirozu, Keiko Nobukuni, Kouta Funakoshi, Taizo Nakamura, Makoto Sumie, Midoriko Higashi, Ken Yamaura

**Affiliations:** a Department of Anesthesiology and Critical Care Medicine, Kyushu University Hospital, Fukuoka, Japan; b Operating Rooms, Kyushu University Hospital, Fukuoka, Japan; c Center for Clinical and Translational Research, Kyushu University Hospital, Fukuoka, Japan; d Department of Anesthesiology and Critical Care Medicine, Graduate School of Medical Sciences, Kyushu University, Fukuoka, Japan.

**Keywords:** anesthesia, memory, randomized controlled trial, remimazolam

## Abstract

**Background:**

Remimazolam, a benzodiazepine ultra-short-acting sedative, has been used in general anesthesia since August 2020. It is used in awake surgeries that require awakening the patient in the middle of the surgery because of its rapid awakening effect as well as antagonistic interactions. If remimazolam has associated anterograde amnesia similar to benzodiazepines, it will have a positive effect on preventing psychological trauma. However, to our knowledge, the effect of remimazolam on anterograde amnesia has not been previously examined.

**Methods/design:**

The aim of this exploratory, open, propofol-controlled, single-center, randomized clinical trial is to examine the effect of remimazolam on postoperative memory retention and delayed regeneration. Seventy patients undergoing breast surgery will be included in the study. The patients will be randomly assigned to receive propofol or remimazolam as sedatives during surgery. The primary endpoint is the number of posters patients remember 24 hours after surgery (among 4 posters shown after awakening from anesthesia) as an assessment of anterograde amnesia. Secondary endpoints are retrograde amnesia, dose of analgesic given from the time the patient returns to the ward until 24 hours after surgery, immediate postoperative pain numerical rating scale scores, and pain numerical rating scale scores 24 hours after leaving the operating room. Recruitment will take place between October 2021 and March 2022 to achieve the target sample size.

**Discussion:**

To our knowledge, this is the first trial designed to examine the effects of remimazolam on postoperative memory retention and delayed regeneration in patients undergoing breast surgery.

**Trial registration:**

This clinical trial was registered at the University Hospital Medical Information Network (UMIN) Center on September 28, 2021 (UMIN-CTR: UMIN000045593).

## 1 Introduction

Remimazolam, a benzodiazepine ultra-short-acting sedative, has been used in general anesthesia in Japan since August 2020; it was first implemented in Japan. This medication has similar characteristics as propofol (eg, it is short acting) and is expected to be used in a similar manner. Clinical trials conducted in Japan have reported that its effect on hemodynamics is less than that of propofol and is considered an easy-to-use sedative in patients with unstable circulation.^[1,2]^

Although anesthetics were originally designed to prevent intraoperative awakening, some surgeries (such as brain surgery under awake conditions) require awakening for medical reasons in the middle of the surgery.^[3]^ Traditionally, propofol has been used in such surgeries because it allows for rapid awakening. However, intraoperative awakening inevitably causes patients discomfort during surgery. If that memory persists postoperatively, it may have a traumatic effect on the patient.

Remimazolam has been used in surgeries that require awakening the patient in the mid-surgery because it allows for rapid awakening (similar to propofol and antagonists). Since remimazolam is a benzodiazepine, it is expected to cause anterograde amnesia (similar to other benzodiazepines)^[4,5]^ and to decrease the discomfort caused by intraoperative awakening; this property is expected to have a positive effect on preventing psychological trauma.

Clinically, we observed that patients have no memory of the immediate postoperative period after general anesthesia with remimazolam. In addition, based on postoperative interviews of patients sedated with remimazolam, we found that all patients had no memory of postoperative pupillometry examination, bed transfer, or conversations occurring during and shortly following sedation. However, we cannot conclude that remimazolam causes anterograde amnesia based on the current evidence because there is no established method to evaluate anterograde amnesia, and our observations are anecdotal and obtained within the scope of our practice. Specifically, this study aims to compare the effects of different sedatives used in general anesthesia on memory retention in postoperative patients and to establish a method to evaluate effects on anterograde amnesia in validation clinical trials.

## 2 Methods

### 2.1 Objectives

The aim of this study is to verify the effects of remimazolam use in general anesthesia on memory retention in postoperative patients and to establish a method to evaluate its effects on anterograde amnesia in validation clinical trials.

### 2.2 Study design

**Figure 1:**
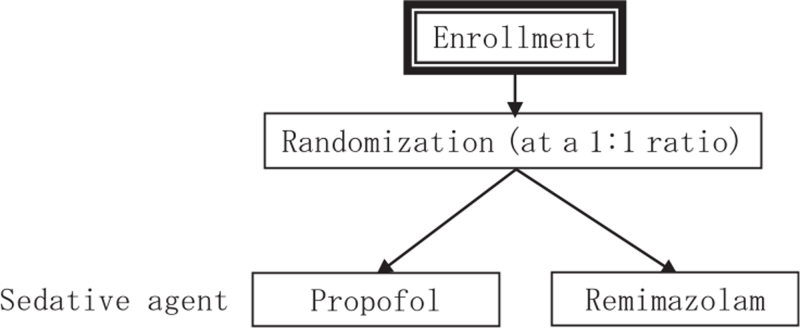
Study design.

The standard protocol items for randomized trials guidelines were followed. The present study is an exploratory, open, propofol-controlled, single-center, randomized controlled trial (RCT) to compare the efficacy and safety of remimazolam with propofol. The study will be conducted by the Department of Anesthesiology and Critical Care Medicine, Kyushu University Hospital. In total, 70 patients aged 20 to 65 years will be randomly assigned to receive remimazolam or propofol as sedative anesthetic agent. The participants will be randomly divided into 2 groups using stratified block randomization. The study design is summarized in Figure 1 and available at URL (https://upload.umin.ac.jp/cgi-open-bin/ctr_e/ctr_view.cgi?recptno=R000052026)).

### 2.3 Investigational drug

Remimazolam is a standard sedative commonly used in clinical practice. It is similar to the benzodiazepine-like drug midazolam, which is also widely used in clinical settings. Remimazolam differs from midazolam in that its metabolites have no pharmacological effects and its sedative effects disappear rapidly upon the discontinuation of administration. The study drug (remimazolam: mundipharma K.K., Tokyo, Japan) is initiated at 6 mg/kg/h, which is changed to 1 mg/kg/h after confirming sleep. During surgery, the dose should be adjusted as required in the range of 0.5 to 2 mg/kg/h with reference to the bispectral index (BIS) value.

The initial dose of control dug (propofol: Maruishi Pharmaceutical Co., Ltd, Osaka, Japan) is 3 μg/mL and is administered using a target-controlled infusion pump and is adjusted with reference to the BIS value.

While the patient is still unconscious, flumazenil should be administered 5 minutes after the surgery ends. Flumazenil is intravenously and slowly administered at an initial dose of 0.2 mg. If the patient's wakefulness level is not sufficiently achieved within 4 minutes after administration, an additional 0.1 mg should be administered. If necessary, 0.1 mg should be administered at 1-minute intervals, up to a total dose of 1 mg.

For concomitant medications, the usual dosage of fentanyl and remifentanil for intraoperative analgesia should be administered. The dosage of remifentanil should not exceed a maximum of 0.5 μg/kg/min. A contraindication that should be considered is that antihistamines should not be administered between the day prior to surgery and the day after surgery.

### 2.4 Inclusion criteria

The following criteria are necessary to meet eligibility requirements for the current study.

(1) Patients must provide written informed consent of their own free will.
(2) Patients must be aged ≥20 years and must be <65 years.
(3) Patients must be undergoing breast surgery under general anesthesia.

In breast surgery cases, intraoperative techniques are relatively uniform, and there are slight individual differences in the degree of invasion. This is because the dosage of analgesics and sedatives does not vary greatly. Although the study will be conducted only in women, there were no reports of differences in memory retention between men and women. Therefore, priority was given to unification of the degree of invasion.

### 2.5 Exclusion criteria

Patients should be excluded if any one of the following criteria apply.

(1) Patients with a history of hypersensitivity to remimazolam or propofol.
(2) Patients with egg or soybean oil allergies, acute angle-closure glaucoma, myasthenia gravis, serious disease complications, or a history of allergies.
(3) Patients with an American Society of Anesthesiologists physical status of ≥IV, shock, or coma.
(4) Patients who are deemed inappropriate for inclusion in the study.
(5) Patients with impaired cognitive function.

### 2.6 Discontinuation criteria

For individual study participants:

If the principal investigator or sub-investigator determines that continuation of the study is not feasible for any of the reasons listed below, the patient will be removed from this study. In such cases, the reason for discontinuation will be explained to the patient as necessary.

(1) Study participants declining to participate in the study or withdrawing their consent.
(2) The principal investigator or subcontractor deciding that it is appropriate to discontinue the study.
(3) The entire study plan being canceled.

Discontinuation, suspension, or termination of the trial:

If the Safety Monitoring Committee decides to stop the trial, the entire trial will be stopped.

### 2.7 Outcome measurements

**Figure 2:**
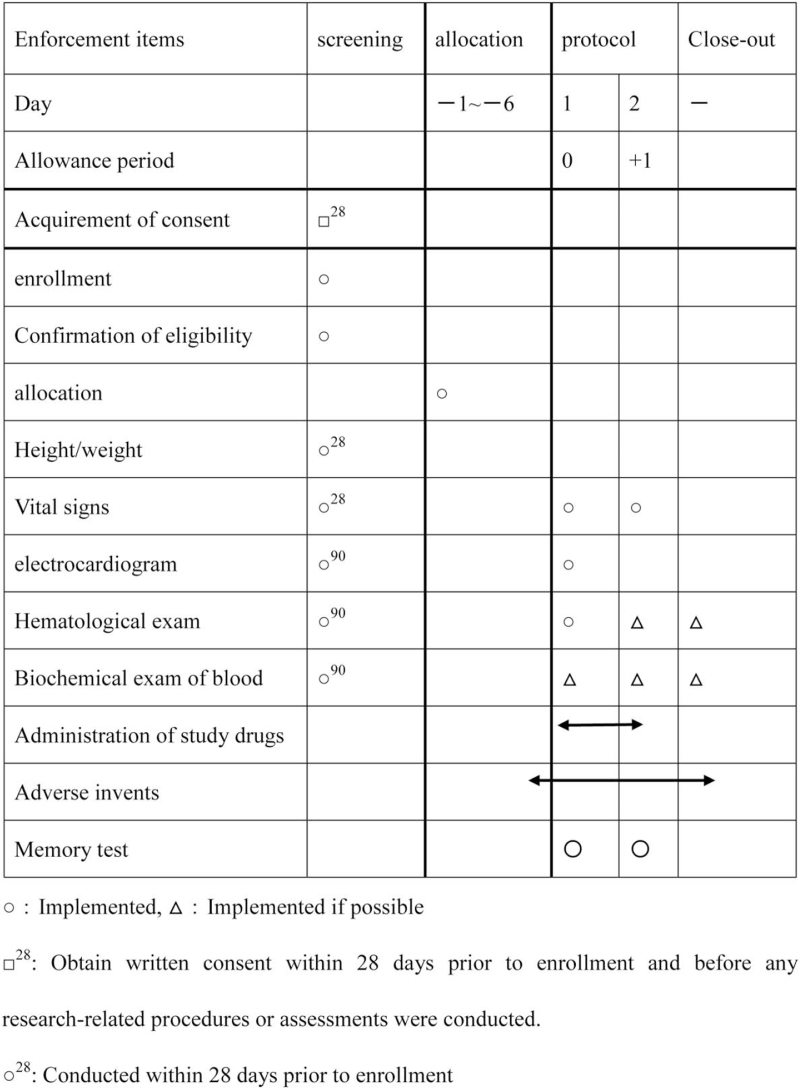
Schedule of enrollment, intervention, and assessment.

The assessment schedule is represented in Figure 2.

#### 2.7.1 Primary endpoint

The primary endpoint to be examined in this RCT is the assessment of anterograde amnesia. This will be examined as the number of posters remembered among 4 posters 24 hours after surgery (see Figure S1, Supplemental Digital Content, which illustrates the pictures) shown after awakening from anesthesia. Specifically, after awakening from anesthesia and being able to say their names, patients are to be shown 4 posters.^[6]^

#### 2.7.2 Secondary endpoints

(1) Retrograde amnesia, based on the number of events remembered from prior to the start of anesthesia – ambulatory entry, bed transfer, vein insertion, BIS monitor attachment to the forehead, and mask fitting – and whether the patient remembers the poster (see Figure S2, Supplemental Digital Content, which illustrates the numeric characters) shown before the induction of anesthesia.
(2) The dose of analgesic medication administered from the time the patient returns to the ward until 24 hours after surgery.
(3) Immediate postoperative pain numerical rating scale (NRS) values (see Figure S3, Supplemental Digital Content, which illustrates the NRS).
(4) Pain NRS scores 24 hours after leaving the operating room.

#### 2.7.3 Safety endpoints

1) Nausea assessed based on the dose of antiemetic medication used immediately after surgery and from the time of returning to the ward until 24 hours after surgery.
2) Time to full awakening.
3) The incidences of hypotension, bradycardia, rhabdomyolysis, and anaphylaxis.

### 2.8 Randomization

An allocation supervisor appointed by the Data Center of the ARO Clinical Research Promotion Department of Clinical and Translational Center has prepared an allocation table for the purpose of randomization. The electronic data capture software used in this study refers to this allocation table for randomly allocating patients. The allocation ratio was set at 1:1. The detailed procedure of random allocation has not been provided to the researchers. The permuted block randomization block method will be used for allocation.

### 2.9 Data collection

Data will be recorded and stored via REDCap software (Nashville, TN) while the study is in progress, and the data will then be exported from the database into statistical software for analysis.^[7,8]^ REDCap, which allows for additional data privacy and security protections compared with other data storage platforms, has an audit trail that records every time a participant or staff member makes changes to any data entered on the website. The data used for this study, although not available in a public repository, will be made available in an anonymized and de-identified form to other researchers upon reasonable request. Data and outcome assessors are blinded.

### 2.10 Sample size

Since this is an exploratory study, a sample size calculation was not performed. The researchers aimed for 70 participants because this was considered a feasible number for enrolment that will provide sufficient information for the confirmatory trial.

There have been no similar studies in the past, and we set the number of patients in each of the 2 groups to 35 cases each; we estimate that this number of patients can be enrolled within approximately 6 months.

### 2.11 Adverse events

All adverse events (AEs) that occur between the day of surgery and the end of day 2 after surgery will be recorded. A serious AE is defined as any adverse reaction resulting in any of the following outcomes: a life-threatening condition, death, or a condition that requires inpatient hospitalization or prolongation of an existing hospitalization and threatens to cause disability. Any serious AE will be documented in the medical records and reported to the Clinical Research Board by the responsible investigator, in accordance with the Japanese governmental regulations. If necessary, the investigators will administer treatment for any AE occurring during the course of the study.

### 2.12 Statistical considerations

The full analysis set is defined as all patients enrolled in this study, excluding cases of serious noncompliance with ethical guidelines for life sciences and medical research involving human subjects, untreated cases, and unobserved cases (ie, complete absence of the primary endpoint). The per-protocol set (PPS) is defined as the population of patients included in the full analysis set who do not meet any of the following conditions:

(1) Patients who do not meet the selection criteria specified in this protocol or who violate the exclusion criteria.
(2) Significant deviations from the study protocol.
(3) Patients who fail to receive sufficient anesthetics (ie, the BIS value during surgery not being lower than that before induction of anesthesia).
(4) Cases of concomitant use of prohibited drugs.

The safety analysis set comprises the population of patients who receive at least 1 dose of the study drug.

#### 2.12.1 Analysis of primary and secondary endpoints

Because the goal of this small exploratory study is to generate appropriately powered large-scale RCTs for future, we will perform descriptive statistics using PPS. After evaluation with descriptive statistics, for additional information, analysis of covariance including the baseline value as a covariate will be performed for comparison of groups. If the distribution is greatly skewed in a blinded review, transformation of values such as logarithmic transformation will be considered. As a sensitivity analysis of the primary endpoint, the same analyses will be performed for the PPS. All analyses will be performed with a two-sided significance level of .1.

#### 2.12.2 Safety analysis

The subject of this analysis will be the safety analysis set. The incidence of each AE (hypotension, bradycardia, rhabdomyolysis, and anaphylaxis) will be compared between the anesthesia groups using the χ-square test or Fisher exact test.

### 2.13 Monitoring

This research will be periodically monitored to ensure that the study is conducted safely and in accordance with the implementation plan as well as to determine if data are being recorded accurately and stored properly.

### 2.14 Ethics approval and consent to participate

This trial is being conducted at Kyushu University in Japan. The study has received approval from the ethics review board at the Kyushu University School of Medicine Hospital Institutional Review Board (IRB approval number: 20212006) and will be conducted in accordance with the World Medical Association Declaration of Helsinki and its later amendments. All participants will be required to sign a written consent form prior to participation. Before consent is obtained, the investigators will provide an oral explanation of the study to each participant. Participation is voluntary, and patients’ privacy will be protected. The participants will be informed of their right to withdraw from this study at any time, and that this will not affect their clinical care. If this study poses a serious health risk to a participant, compensation will be provided. The study results will be published in a peer-reviewed journal and will be presented at national and international conferences.

### 2.15 Funding

No external funders are not pertained to this manuscript. Funding is provided by self-funding, donation to our course, department of Anesthesiology in Kyushu University Hospital. There are no funders associated with this manuscript.

## 3 Discussion

This manuscript presents a protocol for an exploratory, randomized, open, propofol-controlled, single-center clinical trial that will be conducted to investigate the effect of remimazolam on postoperative memory retention and delayed regeneration in breast surgery patients. To our knowledge, this is the first study to elucidate the effect of remimazolam on anterograde amnesia. The study results, if confirmed, will be valuable for bedside physicians because there are few reports on remimazolam administration in clinical settings, especially for general anesthesia.

Anterograde amnesia effects of anesthetics are sometimes useful for patient comfort and can be used in various clinical situations. In contrast, this study will focus on memory. Hence, the cutoff for age was defined at <65 years. Benzodiazepine is associated with retrograde amnesia for a brief period and with respect to visual and event memory.^[9]^ Thus, we evaluated retrograde amnesia as a secondary endpoint. Additionally, when remimazolam is used in future endoscopy examinations at outpatient clinics, time to full awakening (as a safety evaluation) may be an indicator of length of hospital stay. This trial will help inform the development of future large-scale RCTs investigating the effects of remimazolam on postoperative memory retention and will thus help inform research directions, medical guidelines, and medical decision-making.

### 3.1 Trial status

The protocol version presented here is version 1.0 from August 30, 2021. The trial is currently in the participant recruitment stage, which begins on October 13, 2021. Recruitment is expected to be completed by March 31, 2022.

## Acknowledgments

The authors would like to acknowledge Kenji Sakanashi and Saki Hirata for their assistance in making the RED cap.

## Author contributions

**Conceptualization:** Kazuhiro Shirozu, Kouta Funakoshi, Keiko Nobukuni.

**Data curation:** Keiko Nobukuni, Makoto Sumie.

**Formal analysis:** Kouta Funakoshi.

**Investigation:** Kazuhiro Shirozu, Keiko Nobukuni, Makoto Sumie.

**Methodology:** Kazuhiro Shirozu, Keiko Nobukuni, Kouta Funakoshi.

**Project administration:** Kazuhiro Shirozu

**Software:** Taizo Nakamura.

**Writing – original draft:** Kazuhiro Shirozu.

**Writing – review & editing:** Kouta Funakoshi, Midoriko Higashi, Ken Yamaura.

## Supplementary Material

Supplemental Digital Content

## Supplementary Material

Supplemental Digital Content

## Supplementary Material

Supplemental Digital Content
