# Supplementary material for: The effect of remimazolam on postoperative memory retention and delayed regeneration in breast surgery patients: Rationale and design of an exploratory, randomized, open, propofol-controlled, single-center clinical trial: A study protocol
Source: Medicine (Baltimore). 2021 Dec 3;100(48):e27808. doi: 10.1097/MD.0000000000027808 (PMC9191336; doi:10.1097/MD.0000000000027808)
Supplement: Supplemental Digital Content [file medi-100-e27808-s002.docx]

**Supplement 2**
