# Supplementary material for: The effect of remimazolam on postoperative memory retention and delayed regeneration in breast surgery patients: Rationale and design of an exploratory, randomized, open, propofol-controlled, single-center clinical trial: A study protocol
Source: Medicine (Baltimore). 2021 Dec 3;100(48):e27808. doi: 10.1097/MD.0000000000027808 (PMC9191336; doi:10.1097/MD.0000000000027808)
Supplement: Supplemental Digital Content [file medi-100-e27808-s003.docx]

**Supplement 3**

Numerical Rating Scale (NRS)


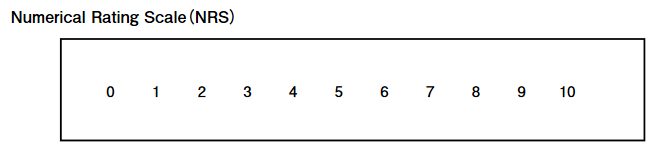


Instructions: Verbally ask, “If zero is no pain at all, and 10 is the worst pain you can think of (i.e., there is no more severe pain), what is your current pain score?” If the answer is 7.5, ask the patient if it is 7 or 8, and align the score to a whole number.
